# Delivery of methoxymorpholinyl doxorubicin by interleukin 2-activated NK cells: effect in mice bearing hepatic metastases

**DOI:** 10.1038/sj.bjc.6690171

**Published:** 1999-03

**Authors:** L Quintieri, A Rosato, N Amboldi, C Vizler, D Ballinari, P Zanovello, D Collavo

**Affiliations:** Department of Oncology and Surgical Sciences, University of Padova, 35128 Padova, Italy; Pharmacia & Upjohn, Discovery Research/Oncology, 20014 Nerviano (MI), Italy

**Keywords:** methoxymorpholinyl doxorubicin, drug delivery, A-NK cell, liver metastases

## Abstract

The possibility of using interleukin 2 (IL-2)-activated natural killer cells (A-NK) to carry methoxymorpholinyl doxorubicin (MMDX; PNU 152243) to liver-infiltrating tumours was explored in mice bearing 2-day established M5076 reticulum cell sarcoma hepatic metastases. In vitro, MMDX was 5.5-fold more potent than doxorubicin against M5076 tumour cells. MMDX uptake by A-NK cells correlated linearly with drug concentration in the incubation medium [correlation coefficient (*r*) = 0.999]; furthermore, as MMDX incorporation was readily reproducible in different experiments, the amount of drug delivered by A-NK cells could be modulated. In vivo experiments showed that intravenous (i.v.) injection of MMDX-loaded A-NK cells exerted a greater therapeutic effect than equivalent or even higher doses of free drug. The increase in lifespan (ILS) following A-NK cell delivery of 53 μg kg^−1^ MMDX, a dosage that is ineffective when administered in free form, was similar to that observed in response to 92 μg kg^−1^ free drug, a dosage close to the 10% lethal dose (ILS 42% vs. 38% respectively). These results correlated with pharmacokinetic studies showing that MMDX encapsulation in A-NK cells strongly modifies its organ distribution and targets it to tissues in which IL-2 activated lymphocytes are preferentially entrapped after i.v. injection. © 1999 Cancer Research Campaign


*British Journal of Cancer* (1999) **79**(7/8), 1067–1073
© 1999 Cancer Research Campaign
Article no. bjoc.1998.0171

### Delivery of methoxymorpholinyl doxorubicin by interleukin 2-activated NK cells: effect in mice bearing hepatic metastases

**L Quintieri[1], A Rosato[1], N Amboldi[2], C Vizler[1], D Ballinari[2], P Zanovello[1] and D Collavo[1]**

[1]Department of Oncology and Surgical Sciences, University of Padova, 35128 Padova, Italy; [2]Pharmacia & Upjohn, Discovery Research/Oncology, 20014 Nerviano (MI), Italy

**Summary** The possibility of using interleukin 2 (IL-2)-activated natural killer cells (A-NK) to carry methoxymorpholinyl doxorubicin (MMDX; PNU 152243) to liver-infiltrating tumours was explored in mice bearing 2-day established M5076 reticulum cell sarcoma hepatic metastases. In vitro, MMDX was 5.5-fold more potent than doxorubicin against M5076 tumour cells. MMDX uptake by A-NK cells correlated linearly with drug concentration in the incubation medium [correlation coefficient (*r*) = 0.999]; furthermore, as MMDX incorporation was readily reproducible in different experiments, the amount of drug delivered by A-NK cells could be modulated. In vivo experiments showed that intravenous (i.v.) injection of MMDX-loaded A-NK cells exerted a greater therapeutic effect than equivalent or even higher doses of free drug. The increase in lifespan (ILS) following A-NK cell delivery of 53 $\mu$g kg$^{-1}$ MMDX, a dosage that is ineffective when administered in free form, was similar to that observed in response to 92 $\mu$g kg$^{-1}$ free drug, a dosage close to the 10% lethal dose (ILS 42% vs. 38% respectively). These results correlated with pharmacokinetic studies showing that MMDX encapsulation in A-NK cells strongly modifies its organ distribution and targets it to tissues in which IL-2 activated lymphocytes are preferentially entrapped after i.v. injection.

**Keywords:** methoxymorpholinyl doxorubicin; drug delivery; A-NK cell; liver metastases

Hepatic metastases from a variety of primary human tumours occur with high frequency, and their presence is usually associated with a low response rate to conventional systemic chemotherapy (Anderson et al, 1994). Efforts to establish innovative drug delivery strategies that improve the efficacy of anti-cancer agents against liver metastases, therefore, are not only desirable but may also produce clinical results more rapidly than the development of new chemical entities (Kim, 1993). Portal vein or hepatic artery infusion of a cytotoxic agent (Anderson et al, 1994), encapsulation of drugs in liposomes (Kim, 1993; Anderson et al, 1994) and liver-targeted erythrocytes (Zocchi et al, 1989) are examples of recently employed attempts to increase the anti-tumour efficacy of chemotherapeutic agents against metastatic liver neoplasms.

We previously explored the possibility of using lymphoid cells, namely cytotoxic T lymphocytes and lymphokine-activated killer cells (LAK) cells, to carry toxins or cytotoxic drugs to the tumour site (Cerundolo et al, 1987; Zanovello et al, 1992; Mandruzzato et al, 1994). As LAK cells are preferentially localized in the host's lungs, liver and spleen following systemic intravenous (i.v.) injection (Lotze et al, 1980; Maghazachi and Fitzgibbon, 1990), we addressed the therapeutic effect of i.v. adoptive transfer of drug-loaded LAK cells in mice bearing lung metastases (Zanovello et al, 1992; Mandruzzato et al, 1994). We found that when ricin or iododoxorubicin (IDX), a lipophilic doxorubicin (DX) derivative, was delivered by LAK cells its efficacy against experimentally induced lung metastases was increased (Zanovello et al, 1992; Mandruzzato et al, 1994).

Methoxymorpholinyl doxorubicin (MMDX; PNU 152243) is a promising, highly lipophilic semisynthetic anthracycline that shows activity against multidrug-resistant cells both in vitro and in vivo and is not cardiotoxic at therapeutic doses (Grandi et al, 1990; Ripamonti et al, 1992; Danesi et al, 1993; Vasey et al, 1995); it is currently undergoing phase I/II clinical trials. This anti-cancer drug is biotransformed in the liver into more cytotoxic metabolites by means of a cytochrome P450-dependent process that can be simulated in vitro by incubating the drug with liver microsomes in the presence of NADPH (Kühl et al, 1993; Lau et al, 1994). This metabolic potentiation by the hepatic microsomal enzymes may contribute to rendering MMDX highly active against liver tumour cell colonies. Preclinical evaluation of MMDX efficacy against hepatic tumours is not yet available, but a phase I clinical study reported regressions in patients with liver metastases from colorectal cancer (Vasey et al, 1995).

Using a mouse liver metastasis model, we evaluated the possible therapeutic advantage of delivering MMDX encapsulated in interleukin 2 (IL-2)-activated natural killer (A-NK) cells. We show here that, in mice bearing hepatic metastases, systemic i.v. adoptive transfer of A-NK cells encapsulating MMDX exerts a greater therapeutic effect than the administration of an equivalent or even higher dose of the drug in free form. Moreover, we observed a significant survival benefit when drug doses that were ineffective in free form were instead delivered by A-NK cells.

*Received 26 September 1997*
*Revised 2 February 1998*
*Accepted 17 June 1998*

*Correspondence to:* P Zanovello, Chair of Immunology, Department of Oncology and Surgical Sciences, University of Padova, Via Gattamelata, 64, 35128 Padova, Italy.

### MATERIALS AND METHODS

### Drugs

MMDX and DX (kindly supplied by Pharmacia & Upjohn as hydrochloride salts) were dissolved in sterile bidistilled water; concentrations were checked spectrophotometrically [MMDX $\lambda_{max}$ 495 nm ($CH_3OH$), E 1% = 173; DX $\lambda_{max}$ 496 nm ($H_2O$), E 1% = 200] and stock solutions (1 mM of MMDX and 0.8 mM of DX) were stored at –20°C. All working solutions were also checked by high-performance liquid chromatography (HPLC) before use.

### Animals and tumour cell line

Seven- to nine-week old inbred female C57BL/6 mice (Charles River, Calco, Italy) were used throughout this study. The mice were fed standard mouse chow, had free access to water and were age matched in individual experiments.

M5076 reticulum cell sarcoma, kindly supplied by Dr R Giavazzi, Mario Negri Institute of Pharmacological Research, Bergamo, Italy, was maintained in vivo as an ascitic tumour in syngenic C57BL/6 mice, with transplants every 3 weeks. M5076 tumour cells preferentially metastasize to the liver after i.v. injection (Hart et al, 1981).

### Monitoring of animals during in vivo experiments

Procedure involving animals and their care were in conformity with institutional guidelines that comply with national and international laws and policies (EEC Council Directive 86/609, OJ L 358, 1, Dec. 12, 1987; NIH Guide for the Care and Use of Laboratory Animals, NIH Publication No. 85-23, 1985).

During in vivo survival experiments, animals in all experimental groups were examined daily for an increase in abdominal volume, decrease in physical activity and other signs of disease in order to identify those expected to become moribund within a short time. Severely ill animals were euthanized by ethyl ether overdose and then autopsied to verify whether their illness had been caused by a large tumour burden. Survival time of each animal was calculated as the number of days elapsed between tumour inoculation and euthanization.

### Generation of A-NK cells

Murine A-NK cells were prepared as previously reported (Gunji et al, 1989). Briefly, single spleen cell suspensions were prepared in RPMI-1640 tissue culture medium (Life Technologies, Paisley, UK) with 3% (v/v) heat-inactivated fetal bovine serum (FBS) (Flow, Irvine, UK). Erythrocytes were lysed by incubation with ammonium chloride–potassium buffer solution. The resulting cells were cultured (37°C, 5% carbon dioxide) with 1000 IU $ml^{-1}$ human recombinant IL-2 (Proleukin, EuroCetus-Chiron, Milan, Italy) in complete medium (CM), which consisted of RPMI-1640 supplemented with 10% (v/v) FBS, 2 mM glutamine, 50 mM 2-mercaptoethanol and antibiotics. After 2 days of incubation, non-adherent cells were discarded and the culture flasks gently washed three times with warm (37°C) CM. The plastic adherent cells were cultured for another 5 days with 1000 U $ml^{-1}$ human recombinant IL-2 in fresh CM. After a short treatment with 0.02% EDTA (w/v) in phosphate-buffered saline (PBS) (Oxoid, Basingstoke, UK) A-NK cells were harvested, washed twice with RPMI-1640 with 3% (v/v) FBS, counted and used; cell recovery rate ranged from 30% to 60% of the seeded spleen cells. The A-NK cells obtained under these experimental conditions were generally 75–90% NK $1.1^+$, and less than 20% $CD3^+$, as determined by flow cytofluorometric analysis with appropriate antibodies, and demonstrated high lytic activity against both LAK-susceptible P815 mastocytoma and M5076 tumour cells (not shown).

### Evaluation of M5076 tumour cell chemosensitivity

MMDX and DX inhibition of M5076 cell growth in vitro was assessed by a slightly modified MTT colorimetric assay as previously described (Kühl et al, 1993). Per cent survival was calculated from the absorbance ($A$) values as follows: survival (%) = $(A)_{tested} - A_{blank})/(A_{untreated\ control} - A_{blank}) \times 100$, with $A_{blank}$ referring to the absorbance of wells that contained only medium and MTT. Drug concentrations that decreased survival by 50% ($IC_{50}$ values) were calculated from semilogarithmic dose–response curves by linear interpolation.

### Evaluation of MMDX uptake and release by A-NK cells

A-NK cells ($5 \times 10^6$ $ml^{-1}$) were incubated with different concentrations of MMDX at 37°C for 30 min in RPMI-1640 supplemented with 3% (v/v) FBS. MMDX influx was stopped by rapidly chilling the tubes to 4°C, and extracellular drug was removed by centrifuging the cells through a layer of horse serum (HS) (Gibco, Life Technologies, Paisley, UK). Cells were then washed once with ice-cold RPMI-1640, aliquoted, centrifuged and their drug content was determined.

To evaluate in vitro drug release, cells ($5 \times 10^6$ $ml^{-1}$) were incubated with 600 nM MMDX at 37°C for 30 min, processed as above, aliquoted, resuspended in drug-free HS and incubated at 37°C for various fixed time intervals. Samples were then rapidly chilled to 4°C, washed once with ice-cold RPMI 1640, centrifuged and their MMDX content evaluated. Each determination was made in quadruplicate.

The amount of intracellular MMDX was determined by HPLC as previously reported for IDX (Mandruzzato et al, 1994) and expressed as ng MMDX $10^{-6}$ cells.

### Induction and therapy of liver metastases

Weight-matched mice were injected with M5076 cells ($5 \times 10^4$ viable cells per mouse) in their tail vein. Two days later, the animals were weighed and randomly assigned to one of the following protocols: i.v. injection of in vitro MMDX-treated A-NK cells; i.v. injection of different amounts of free MMDX in a volume of 0.2 ml PBS; or i.v. injection of only PBS (control mice). In some experiments, an additional experimental group received an i.v. injection of untreated A-NK cells. MMDX doses were based on mean body weight (s.d. never exceeded 0.50 g). The number of MMDX-treated A-NK cells injected varied from 15 to $30 \times 10^6$ per mouse, depending on the number of cells recovered from the different cultures; the drug concentration in the incubation medium thus ranged from 200 to 600 nM in order to load the A-NK cells with the desired amount of MMDX. For every batch of MMDX-loaded cells, an aliquot was evaluated for MMDX content by HPLC. In one set of experiments, mice were killed 21 days after the tumour cell injection; livers were harvested, rinsed in

 © Cancer Research Campaign 1999

water and fixed in Bouin's solution; surface metastases were then counted with the aid of a dissecting microscope. A second set of experiments measured survival time after tumour injection/treatment as described above. Treatment efficacy was evaluated by comparing median survival time (MST) in the treated and control groups, and expressed as ILS as follows:

$$ILS(\%) = (100 \times \text{MST of treated mice/MST of control mice}) - 100$$

The MST of control animals ranged from 24 to 30 days. Treatment was considered ineffective when ILS < 20%.

### Bone marrow toxicity

MMDX was administered either as free drug or encapsulated in A-NK cells to weight-matched healthy mice on a mean body weight basis. Retro-orbital sinus blood samples were collected on days 3, 7 and 11 following drug administration (six mice per experimental group), diluted with 10 mM EDTA, and counted with a haemocytometer after lysis of red blood cells with acidified methyl violet solution [5% acetic acid (v/v)]. White blood cell (WBC) counts were expressed as a percentage of mean value obtained from control mice that received PBS only.

### Drug biodistribution studies

MMDX was administered i.v. either as free drug or encapsulated in A-NK cells to weight-matched mice on a mean body weight basis. At 15, 90 and 150 min, three mice in each treatment group were sacrificed by excess ethyl ether anaesthesia. The liver, spleen and lungs were rapidly excised, thoroughly washed in 0.9% sodium chloride solution and frozen in liquid nitrogen. MMDX was extracted as follows: 2–2.5 ml of distilled water was added to the samples, which were homogenized using an Ultra Turrax apparatus; 0.5 ml of 33% silver nitrate was added to the homogenate, which was then mixed vigorously for 20 min at 4°C; 5 ml of ethyl acetate–*n*-butanol (9:1) was added, and the mixture was further shaken for 10 min at 4°C; samples were then spun at 6000 r.p.m. for 10 min and the organic phase was recovered. The extraction procedure was repeated twice, and the two resulting organic phases were dried in a Braun alpha 4 evaporator. Dried samples were recovered with 0.5 ml of methanol, sonicated for 30 s and spun at 13 000 ***g*** for 10 min at 4°C. The amount of MMDX in the supernatants was evaluated by HPLC analysis as previously reported for IDX (Mandruzzato et al, 1994) and expressed as ng of MMDX per g of tissue. The mean recovery of the drug from different organs ranged from 67% to 87%, with 95% reproducibility.

### Statistical analysis

Statistical analysis was performed using the GraphPad InStat computer program (GraphPad Software, San Diego, CA, USA) and the Mann–Whitney *U*-test.

### RESULTS

### MMDX uptake and release by A-NK cells

Preliminary experiments compared M5076 tumour cell sensitivity to MMDX and DX; cells were incubated with different drug concentrations for 48 h at 37°C, and cell survival was evaluated by

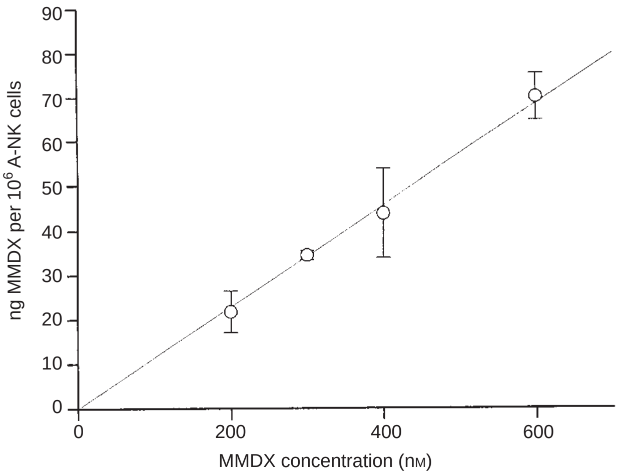

**Figure 1** MMDX uptake by A-NK cells as a function of exposure concentration. Cells ($5 \times 10^6$ $ml^{-1}$) were incubated with different concentrations of MMDX for 30 min at 37°C. After rapid chilling at 4°C, cells were harvested, centrifuged through a layer of HS and washed once with RPMI-1640. MMDX intracellular content was evaluated by HPLC as described in Materials and methods. Each determination was made in quadruplicate. Points, mean of at least three separate experiments; bars, s.d. The line represents the linear regression equation. Correlation coefficient was 0.999

an MTT colorimetric assay. The potency ratio (ratio of the $IC_{50}$ value for DX vs. the $IC_{50}$ value for MMDX) was 5.5 (not shown). This result agrees with reports regarding the in vitro sensitivity of other murine and human tumour cell lines to MMDX and DX (Grandi et al, 1990; Kühl et al, 1993; Lau et al, 1994).

The kinetics of MMDX uptake by A-NK cells was biphasic, with an initial phase of rapid uptake (first 15 min) followed by a steady-state phase (not shown). By fixing the incubation time at 30 min, a linear relationship between the MMDX concentration in the incubation medium and its uptake by A-NK cells emerged (Figure 1). Moreover, the amount of MMDX incorporated by the A-NK cells was highly reproducible in several experiments, probably because of the homogeneity in the size of A-NK cells (Melder et al, 1988; Bouwens et al, 1992). Thus, by varying the MMDX concentration in the incubation medium during cell loading, we could modify the amount of drug delivered by A-NK cells to the tumour-bearing animals.

Analysis of the time course of drug efflux from MMDX-treated A-NK cells revealed that cellular MMDX contents decreased quickly to about 55% within 15 min post incubation; this initial phase of rapid efflux was followed by a second one in which the efflux rate was much slower. A representative experiment is shown in Figure 2. We observed a similar biphase kinetics of drug efflux from IDX-loaded standard LAK cells (Mandruzzato et al, 1994).

### Encapsulation in A-NK cells increases anti-tumour activity of MMDX

The anti-tumour activity of MMDX administered via A-NK cells or in free form was evaluated in terms of tumour reduction and animal survival. In the first set of experiments, the mice were sacrificed on day 21, and the surface hepatic tumour nodules were counted. Mice that had received MMDX delivered by A-NK cells had significantly fewer nodules than mice administered an equivalent amount of free drug. A representative experiment is reported

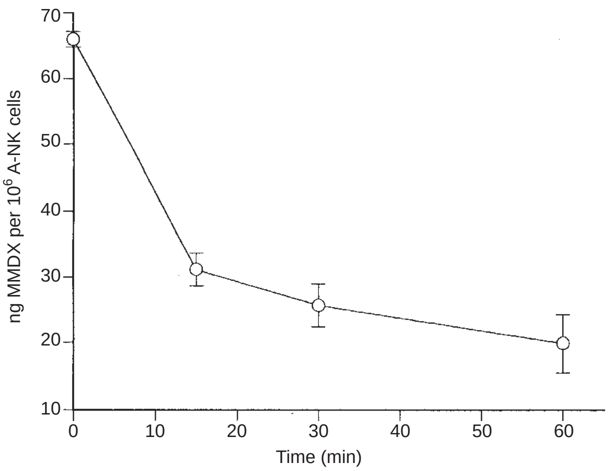

**Figure 2** Time course of drug efflux from MMDX-loaded A-NK cells. Cells ($5 \times 10^6$ $ml^{-1}$) were incubated with 600 nM MMDX for 30 min at 37°C. After rapid chilling at 4°C, cells were harvested, centrifuged, washed once with RPMI-1640 and then resuspended in drug-free HS. At each indicated time-point, four samples were processed to evaluate their MMDX content by HPLC as described in Materials and methods. Points, mean; bars, s.d.

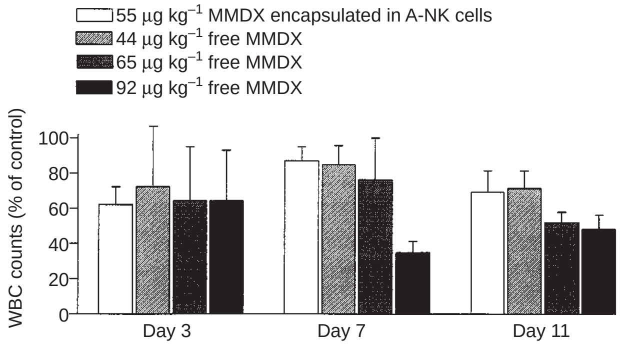

**Figure 3** Myelosuppression studies in healthy C57BL/6 mice given free or A-NK cell-bound MMDX. On day 0, mice received a single i.v. injection of PBS, free MMDX or $27 \times 10^6$ A-NK cells pretreated with 300 nM MMDX for 30 min at 37°C. Samples of retro-orbital sinus blood obtained on days 3, 7 and 11 were processed as described in Materials and methods. WBC counts were expressed as a percentage of values obtained from control animals. Columns, mean ($n$ = 6); bars, s.d.

in Table 1. Treatment with 52 µg kg$^{-1}$ MMDX encapsulated in A-NK cells induced a significantly greater reduction in the number of tumour nodules than the administration of 51 µg kg$^{-1}$ free drug; this reduction was similar to that observed in animals receiving 92 µg kg$^{-1}$ MMDX in free form, a dose close to 10% lethal dose ($LD_{10}$). Furthermore, unlike the animals that received 51 µg kg$^{-1}$ MMDX in free form, 90% of the mice treated with MMDX-loaded A-NK cells were free of visible liver metastases on day 21.

No tumour reduction was observed in experiments in which mice bearing 2-day established M5076 hepatic metastases were injected i.v. with 15–30 × $10^6$ untreated A-NK cells without concomitant injection of IL-2 (not shown).

To determine whether the reduction in the number of liver metastases after adoptive transfer of MMDX-loaded A-NK cells translated into a prolonged survival time, the MSTs of the experimental groups receiving either free or cell-associated MMDX were compared with that of the control group, and treatment efficacy was expressed as per cent ILS. Some experiments included an additional test group consisting of mice receiving untreated A-NK cells alone. As shown in Table 2, treatment with MMDX-loaded A-NK cells was more effective in prolonging survival than the administration of an equivalent or even greater amount of free drug. As seen in experiment II (Table 2), 52 µg kg$^{-1}$ free MMDX was ineffective, but 53 µg kg$^{-1}$ MMDX delivered by A-NK cells granted an ILS equivalent to that afforded by 92 µg kg$^{-1}$ free drug. The administration of untreated A-NK cells or very low doses of A-NK cell-bound or free MMDX did not modify survival of tumour-bearing mice (experiment I, Table 2).

To determine the effects of free or A-NK cell-associated MMDX on haematopoiesis, WBC counts were examined in healthy mice on days 3, 7 and 11 following drug administration. At all investigated time points, WBC counts of mice treated with 55 µg kg$^{-1}$ A-NK cell-bound MMDX were only moderately depressed (Figure 3). In contrast, mice receiving 92 µg kg$^{-1}$ free drug showed a marked myelosuppression on days 7 and 11 (65% and 53% depression of WBC counts respectively).

### In vivo MMDX distribution is modified by encapsulation in A-NK cells

To investigate whether the therapeutic effect observed in mice receiving MMDX-loaded A-NK cells was related to an increased drug uptake by the liver, we compared the tissue distribution of MMDX injected in free form with that of MMDX encapsulated in A-NK cells. Mice were injected with either 58 µg kg$^{-1}$ MMDX encapsulated in A-NK cells, or 80 µg kg$^{-1}$ free MMDX, the lowest drug dose conferring a similar therapeutic effect. Moreover, a group of mice also received 150 µg kg$^{-1}$ free MMDX, a toxic dose producing 100% mortality (not shown). At 15, 90 and 150 min after i.v. injection, three animals per group were killed and drug

**Table 1** Effect of MMDX on M5076 reticulum cell sarcoma growth in the liver

| Group | Treatment[a] | MMDX dose (µg kg$^{-1}$) | Liver metastasis-bearing mice | Median no. (range) of liver metastases | Statistical comparison of metastasis number with group[b] |
|---|---|---|---|---|---|
| A | PBS (control) | – | 10/10 | 137.5 (47–> 250) | B ($P$ < 0.05); C, D, E ($P$ < 0.0001) |
| B | Free MMDX | 41 | 10/10 | 16.5 (8–135) | |
| C | Free MMDX | 51 | 9/10 | 3.5 (0–12) | D ($P$ = 0.001); E ($P$ = 0.0003) |
| D | Free MMDX | 92 | 2/9[c] | 0 (0–1) | E ($P$ = 0.66) |
| E | MMDX-loaded A-NK cells[d] | 52 | 1/10 | 0 (0–1) | |

[a]On day 0, C57BL/6 mice were injected i.v. with $5 \times 10^4$ M5076 tumour cells. Two days later, they were weighed, randomly assigned to an experimental group ($n$ = 10) and received i.v. MMDX either in free form or encapsulated in A-NK cells; control group mice received only PBS. The mice were killed on day 21, and liver metastatic nodules counted with the aid of a dissecting microscope. [b]Two tailed Mann–Whitney test. [c]One mouse out of ten died on day 7. [d]$30 \times 10^6$ A-NK cells pretreated with 300 nM MMDX for 30 min at 37°C.

 *© Cancer Research Campaign 1999*

**Table 2** Effect of MMDX on life span in mice bearing M5076 hepatic metastases

| | Group | Treatment[a] | MMDX dose (μg kg$^{-1}$) | ILS[b] (%) | Statistical comparison with group[c] |
|---|---|---|---|---|---|
| Experiment I | A | PBS (control) | – | | B (*P* = 0.97); C (*P* = 0.85); D (*P* = 0.24); E (*P* = 0.43) |
| | B | Untreated A-NK cells[e] | – | 0 | |
| | C | Free MMDX | 28 | 0 | E (*P* = 0.11) |
| | D | Free MMDX | 55 | 13[d] | E (*P* = 0.73) |
| | E | MMDX-loaded A-NK cells[e] | 21 | 10[d] | |
| Experiment II | F | PBS (control) | – | | G (*P* = 0.07); H (*P* = 0.012); I (*P* = 0.0009); J (*P* = 0.001) |
| | G | Free MMDX | 41 | 10[d] | |
| | H | Free MMDX | 52 | 17[d] | J (*P* = 0.005) |
| | I | Free MMDX | 92 | 38 | J (*P* = 0.94) |
| | J | MMDX-loaded A-NK cells[f] | 53 | 42 | |
| Experiment III | K | PBS (control) | – | | L, M, N (*P* < 0.0001) |
| | L | Free MMDX | 75 | 48 | N (*P* = 0.0034); M (*P* = 0.97) |
| | M | Free MMDX | 84 | 50 | N (*P* = 0.0044) |
| | N | MMDX-loaded A-NK cells[g] | 76 | 70 | |

[a]On day 0, C57BL/6 mice were injected i.v. with 5 × 10$^4$ M5076 tumour cells. Two days later they were weighed, randomly assigned to an experimental group (*n* = 10–15) and received i.v. injection of PBS, untreated A-NK cells, free MMDX or MMDX-treated A-NK cells. The animals were observed daily in order to determine their survival time. [b]Calculated as (100 × MST of treated mice/MST of control mice) –100. [c]Two-tailed Mann–Whitney test. [d]Treatment was considered ineffective when ILS <20%. [e]21 × 10$^6$ A-NK cells incubated in the presence (group E) or absence (group B) 300 nM MMDX for 30 min at 37°C. [f]27.5 × 10$^6$ A-NK cells pretreated with 300 nM MMDX for 30 min at 37°C. [g]22.5 × 10$^6$ A-NK cells pretreated with 500 nM MMDX for 30 min at 37°C.

concentration in liver, spleen and lungs was determined by HPLC.

Results are summarized in Figure 4. Fifteen minutes after treatment, drug levels in the livers of mice injected with 58 μg kg$^{-1}$ MMDX encapsulated in A-NK cells were very similar to those observed in animals treated with 80 μg kg$^{-1}$ free drug, and lower than those in mice receiving 150 μg kg$^{-1}$ free drug. At 90 min, however, levels in mice receiving MMDX encapsulated in A-NK cells were much higher than those in mice administered 80 μg kg$^{-1}$ free MMDX, and close to those observed in mice treated with 150 μg kg$^{-1}$ free drug. Similarly, at 150 min, drug levels were higher in mice receiving 58 μg kg$^{-1}$ MMDX via A-NK cells, compared with 80 μg kg$^{-1}$ in free form.

MMDX concentrations in the spleens of animals receiving MMDX-loaded A-NK cells were very low at 15 min after treatment, and less than those detected in mice injected with free drug. However, spleen drug concentrations progressively increased in animals treated with MMDX-loaded A-NK cells, and after 150 min were higher than those observed in mice treated with 150 μg kg$^{-1}$ free MMDX.

The distribution of MMDX was quite different in lungs; 15 min after injection its level in mice that received MMDX-loaded A-NK cells was about 1.7-fold higher than that observed in animals injected with 150 μg kg$^{-1}$ free MMDX. This high organ concentration rapidly decreased, and at 150 min after injection reached a value similar to that observed in animals receiving 80 μg kg$^{-1}$ free MMDX.

### DISCUSSION

We previously demonstrated that i.v. injection of IDX-loaded LAK cells exerted a greater therapeutic effect than an equivalent amount of free drug in mice bearing experimentally induced lung metastases; this effect was largely due to the persistent high drug levels reached in the lungs following delivery by LAK cells (Mandruzzato et al, 1994). The distinctive feature of this chemo-immunotherapeutic approach is that tissue drug concentrations are determined mainly by the distribution pattern of the transferred cytotoxic cells, which are primarily trapped in the lungs following

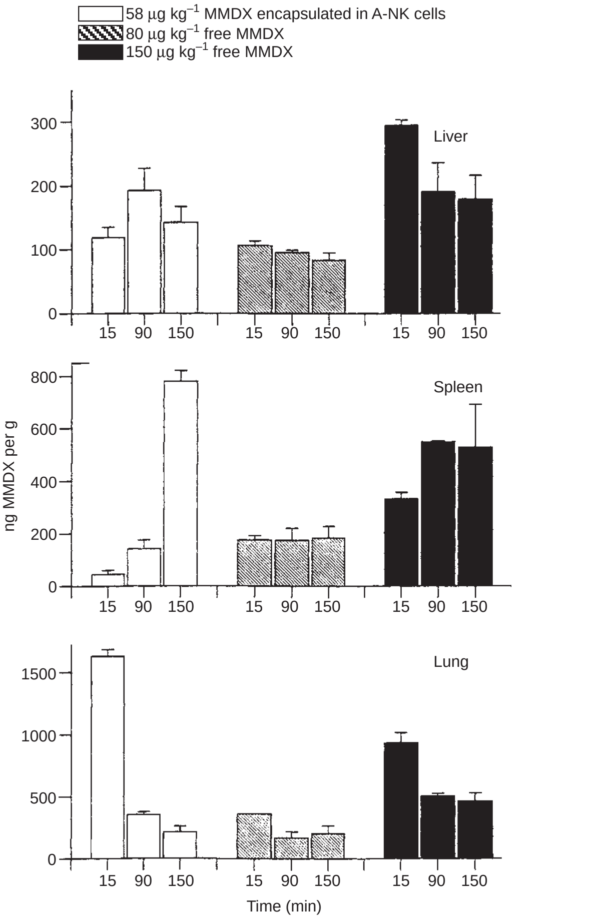

**Figure 4** Drug levels in liver, spleen and lung after i.v. injection of free MMDX or MMDX-loaded A-NK cells. Weight-matched C57BL/6 mice received i.v. MMDX in free form or encapsulated in A-NK cells (15.5 × 10$^6$ A-NK cells pretreated with 600 nM MMDX for 30 min at 37°C). At the different time points, three mice per experimental group were sacrificed; the liver, spleen and the lungs were rapidly excised, weighed and processed for drug content evaluation as described in Materials and methods. Columns, mean of three mice per experimental group; bars, s.d.

© Cancer Research Campaign 1999 

systemic i.v. injection and then migrate to the liver and spleen (Lotze et al, 1980; Maghazachi and Fitzgibbon, 1990). On the basis of these findings, the present study explored the possibility of curing tumours localized in the liver by administering via A-NK cells a novel anthracycline, MMDX, which is characterized by a wide spectrum of anti-neoplastic activity and whose enzymatic transformation in the liver produces highly cytotoxic metabolites.

A-NK cells, initially referred to as adherent lymphokine-activated killer cells, are cytotoxic lymphocytes endowed with a large granular lymphocyte morphology, an NK phenotype and a higher in vitro and in vivo anti tumour activity as compared with conventional LAK cells (Melder et al, 1988; Gunji et al, 1989; Schwarz et al, 1989). Furthermore, in vitro expansion of A-NK cells is generally faster than that of LAK cells, despite a strong interindividual variation in the proliferation rate, particularly in A-NK cells generated from peripheral blood lymphocytes of patients with cancer (Melder et al, 1988). By using A-NK cells, we could overcome the major drawback of the previously proposed experimental procedure, i.e, the high variability in drug incorporation by in vitro IL-2-activated lymphocytes (Mandruzzato et al, 1994). Indeed, the higher homogeneity in the size of the A-NK cells obtained at the end of the culture period resulted in a high reproducibility of MMDX uptake and a linear correlation between drug concentration in the incubation medium and its accumulation in A-NK cells (Figure 1). Therefore, by using A-NK cells, the amount of MMDX delivered by carrier cells to tumour-bearing animals could be modulated with high precision.

The adoptive transfer of MMDX-loaded A-NK cells in mice bearing hepatic metastases led to a drastic decrease in the number of tumour nodules. Indeed, a count of the visible liver metastases 21 days after the tumour cell injection disclosed that treatment with 50–60 μg $kg^{-1}$ MMDX delivered by A-NK cells exerted a strong therapeutic effect. As seen in the representative experiment reported in Table 1, only one of ten mice receiving 52 μg $kg^{-1}$ MMDX via A-NK cells developed a single neoplastic nodule in the liver, whereas nine of ten mice receiving a similar amount of free MMDX exhibited single or even multiple metastases; a similar reduction in the number of hepatic tumour nodules was achieved in response to a dose of free MMDX that was about twice that delivered by A-NK cells, and close to $LD_{10}$. Moreover, in mice receiving MMDX-loaded A-NK cells, the reduction in the number of metastatic liver nodules observed on day 21 translated into a significantly greater prolongation of the survival time, in comparison with mice injected with a similar amount of free drug.

The critical finding of this study is that a significant survival benefit is obtained when drug doses that are ineffective or marginally effective in free form are instead delivered by A-NK cells. Pharmacokinetic studies explain this therapeutic effect by showing that MMDX encapsulation in A-NK cells alters its tissue distribution, and targets it to those organs in which A-NK cells are preferentially retained after i.v. injection. At all the time points investigated, drug levels in the liver tissue of mice receiving 58 μg $kg^{-1}$ MMDX encapsulated in A-NK cells were similar to or even higher than those of mice receiving 80 μg $kg^{-1}$ free MMDX, which is the lowest free drug dose able to grant a therapeutic effect in terms of tumour reduction, and also an ILS similar to that observed in response to 50–60 μg $kg^{-1}$ MMDX delivered via A-NK cells. The accumulation of MMDX-loaded A-NK cells in the liver, and the subsequent slow MMDX efflux may be mainly responsible for the observed enhanced anti-tumour effect. The slow drug release from MMDX-loaded A-NK cells entrapped in the spleen, an organ whose venous blood is drained through the portal vein system, may also contribute to the therapeutic effect in this tumour model. Indeed, considering the high drug level reached in the spleen at 150 min after injection, this contribution might be substantial.

That the intrinsic anti-tumour activity of A-NK cells might contribute to the observed therapeutic effect after administration of MMDX-loaded A-NK cells could be excluded, as a single i.v. infusion of untreated A-NK cells at the doses employed in this study (15–30×$10^6$ cells per mouse), without concomitant administration of IL-2, neither decreased the number of liver metastatic nodules on day 21 (not shown) nor affected the survival of tumour-bearing mice (Table 2, experiment I). Furthermore, the in vitro cytolytic activity of MMDX-treated A-NK cells 4 h after incubation with the drug was only about 40% of controls, albeit no cell membrane alterations, as judged by the eosin dye exclusion test, were observed at this time (not shown).

Myelosuppression represents the dose-limiting toxicity of MMDX (Vasey et al, 1995). Experiments in which the bone marrow toxicity of 55 μg $kg^{-1}$ MMDX delivered by A-NK cells was compared with that of 92 μg $kg^{-1}$ free MMDX, a drug dose that showed a similar therapeutic efficacy in hepatic metastases-bearing mice, disclosed that the former induced only a moderate decrease of peripheral WBC counts at all the investigated time points; 92 μg $kg^{-1}$ free MMDX instead produced a marked haematological toxicity resulting in a 65% depression of WBC counts on day 7 (Figure 3).

In conclusion, by means of our delivery system, a therapeutic effect against murine hepatic metastases could be achieved at an MMDX dosage that did not produce significant myelotoxicity. This approach could possibly increase the clinical utility of this chemotherapeutic agent in patients with hepatic neoplasms.

### ACKNOWLEDGEMENTS

We wish to thank Dr C Geroni and Dr M Grandi for critical reading of the manuscript, and Mrs P Segato for expert help in its preparation. We also thank Mr V Barbieri for the competent technical assistance and Dr Fumagalli, EuroCetus-Chiron, Milan, Italy, for generously providing human recombinant IL-2. This study was supported by the National Research Council of Italy, Target Project ACRO, the Associazione Italiana per la Ricerca sul Cancro (AIRC), and grants 60% and 40% from the Italian Ministry of Public Education. L Quintieri and C Vizler are supported by fellowships from AIRC. A Rosato is supported by a fellowship from the Istituto Superiore di Sanità-Progetto AIDS.

### REFERENCES

Anderson JH, Warren W and McArdle CS (1994) Clinical pharmacokinetic advantages of new drug delivery methods for the treatment of liver tumours. *Clin Pharmacokinet* **27**: 191–201

Bouwens L, Narayani I and Wisse E (1992) High deformability and motility of lymphokine-activated killer cells *in vitro* and *in vivo*. *J Leukoc Biol* **51**: 214–219

Cerundolo V, Zanovello P, McIntosh D, Fabbris R, Davies AJS and Collavo D (1987) Temporary inhibition of Moloney-murine sarcoma virus (M-MSV) induced-tumours by adoptive transfer of ricin-treated T-lymphocytes. *Br J Cancer* **55**: 413–419

Danesi R, Agen C, Grandi M, Nardini V, Bevilacqua G and Del Tacca M (1993) 3′-deamino-3′-(2-methoxy-4-morpholinyl)-doxorubicin (FCE 23762): a new anthracycline derivative with enhanced cytotoxicity and reduced cardiotoxicity. *Eur J Cancer* **29A**: 1560–1565

© Cancer Research Campaign 1999

Grandi M, Pezzoni G, Ballinari D, Capolongo L, Suarato A, Bargiotti A, Faiardi D and Spreafico F (1990) Novel anthracycline analogs. *Cancer Treat Rev* **17**: 133–138

Gunji Y, Vujanovic NL, Hiserodt JC, Herberman RB and Gorelik E (1989) Generation and characterization of purified adherent lymphokine-activated killer cells in mice. *J Immunol* **142**: 1748–1754

Hart IR, Talmadge JE and Fidler IJ (1981) Metastatic behavior of a murine reticulum cell sarcoma exhibiting organ-specific growth. *Cancer Res* **41**: 1281–1287

Kim S (1993) Liposomes as carriers of cancer chemotherapy. Current status and future prospects. *Drugs* **46**: 618–638

Kühl J, Duran GE, Chao NJ and Sikic BI (1993) Effects of the methoxymorpholino derivative of doxorubicin and its bioactivated form versus doxorubicin on human leukemia and lymphoma cell lines and normal bone marrow. *Cancer Chemother Pharmacol* **33**: 10–16

Lau DHM, Duran GE, Lewis AD and Sikic BI (1994). Metabolic conversion of methoxymorpholinyl doxorubicin: from a DNA strand breaker to a DNA cross-linker. *Br J Cancer* **70**: 79–84

Lotze MT, Line BR, Mathisen DJ and Rosenberg SA (1980) The *in vivo* distribution of autologous human and murine lymphoid cells grown in T cell growth factor (TCGF): implication for the adoptive immunotherapy of tumors. *J Immunol* **125**: 1487–1493

Maghazachi AA and Fitzgibbon L (1990) Fate of intravenously administered rat lymphokine-activated killer cells labeled with different markers. *Cancer Immunol Immunother* **31**: 139–145

Mandruzzato S, Rosato A, Bronte V, Zanovello P, Amboldi N, Ballinari D and Collavo D (1994) Adoptive transfer of lymphokine-activated killer cells loaded with 4′-deoxy-4′-iododoxorubicin: therapeutic effect in mice bearing lung metastases. *Cancer Res* **54**: 1016–1020

Melder RJ, Whiteside TL, Vujanovic NL, Hiserodt JC and Herberman RB (1988) A new approach to generating antitumor effectors for adoptive immunotherapy using human adherent lymphokine-activated killer cells. *Cancer Res* **48**: 3461–3469

Ripamonti M, Pezzoni G, Pesenti E, Pastori A, Farao M, Bargiotti A, Suarato A, Spreafico F and Grandi M (1992) In vivo anti-tumour activity of FCE 23762, a methoxymorpholinyl derivative of doxorubicin on doxorubicin-resistant tumour cells. *Br J Cancer* **65**: 703–707

Schwarz RE, Vujanovic NL and Hiserodt JC (1989) Enhanced antimetastatic activity of lymphokine-activated killer cells purified and expanded by their adherence to plastic. *Cancer Res* **49**: 1441–1446

Vasey PA, Bisset D, Strolin-Benedetti M, Poggesi I, Breda M, Adams L, Wilson P, Pacciarini MA, Kaye SB and Cassidy J (1995) Phase I clinical and pharmacokinetic study of 3′-deamino-3′-(2-methoxy-4-morpholinyl)doxorubicin (FCE 23762). *Cancer Res* **55**: 2090–2096

Zanovello P, Rosato A, Bronte V, Mandruzzato S, Cerundolo V and Collavo D (1992) Antitumor efficacy of lymphokine-activated killer cells loaded with ricin against experimentally induced lung metastases. *Cancer Immunol Immunother* **35**: 27–32

Zocchi E, Tonetti M, Polvani C, Guida L, Benatti U and De Flora A (1989) Encapsulation of doxorubicin in liver-targeted erythrocytes increases the therapeutic index of the drug in a murine metastatic model. *Proc Natl Acad Sci USA* **86**: 2040–2044

*© Cancer Research Campaign 1999*
